# Identifying the “Mushroom of Immortality”: Assessing the *Ganoderma* Species Composition in Commercial Reishi Products

**DOI:** 10.3389/fmicb.2018.01557

**Published:** 2018-07-16

**Authors:** Andrew L. Loyd, Brantlee S. Richter, Michelle A. Jusino, Camille Truong, Matthew E. Smith, Robert A. Blanchette, Jason A. Smith

**Affiliations:** ^1^School of Forest Resources and Conservation, University of Florida, Gainesville, FL, United States; ^2^Department of Plant Pathology, University of Florida, Gainesville, FL, United States; ^3^Instituto de Biología, Universidad Nacional Autónoma de México (UNAM), Mexico City, Mexico; ^4^Department of Plant Pathology, University of Minnesota, St. Paul, MN, United States

**Keywords:** reishi, lingzhi, *Ganoderma lucidum*, dietary supplements, Polyporales

## Abstract

Species of *Ganoderma*, commonly called reishi (in Japan) or lingzhi (in China), have been used in traditional medicine for thousands of years, and their use has gained interest from pharmaceutical industries in recent years. Globally, the taxonomy of *Ganoderma* species is chaotic, and the taxon name *Ganoderma lucidum* has been used for most laccate (shiny) *Ganoderma* species. However, it is now known that *G. lucidum* sensu stricto has a limited native distribution in Europe and some parts of China. It is likely that differences in the quality and quantity of medicinally relevant chemicals occur among *Ganoderma* species. To determine what species are being sold in commercially available products, twenty manufactured products (e.g., pills, tablets, teas, etc.) and seventeen grow your own (GYO) kits labeled as containing *G. lucidum* were analyzed. DNA was extracted, and the internal transcribed spacer (ITS) region and translation elongation factor 1-alpha (*tef1α*) were sequenced with specific fungal primers. The majority (93%) of the manufactured reishi products and almost half of the GYO kits were identified as *Ganoderma lingzhi*. *G. lingzhi* is native to Asia and is the most widely cultivated and studied taxon for medicinal use. Illumina MiSeq sequencing of the ITS1 region was performed to determine if multiple *Ganoderma* species were present. None of the manufactured products tested contained *G. lucidum* sensu stricto, and it was detected in only one GYO kit. *G. lingzhi* was detected in most products, but other *Ganoderma* species were also present, including *G. applanatum, G. australe, G. gibbosum, G. sessile*, and *G. sinense*. Our results indicate that the content of these products vary and that better labeling is needed to inform consumers before these products are ingested or marketed as medicine. Of the 17 GYO kits tested, 11 kits contained *Ganoderma* taxa that are not native to the United States. If fruiting bodies of exotic *Ganoderma* taxa are cultivated, these GYO kits will likely end up in the environment. The effects of these exotic species to natural ecosystems needs investigation.

## Introduction

*Ganoderma* is a large and diverse, globally distributed genus of wood decay fungi that includes species that cause white rot on a variety of tree species. In addition, practitioners of Eastern traditional medicine have prescribed the use of laccate (shiny) *Ganoderma* species, commonly referred to as “reishi” or “lingzhi,” as a preventative anti-inflammatory treatment or to enhance immunity (Wang et al., 2012; Hennicke et al., 2016). In Asian countries, reishi (this term will be used in this paper to refer to both reishi and lingzhi) products have been used for over 2000 years, and *Ganoderma* has been revered as “the mushroom of immortality” (Stamets, 2000). Reishi is a focal point in ancient Chinese and Japanese artwork and has been associated with royalty, wisdom, sexual prowess, and eternal life (Stamets, 2000). According to Chinese and American pharmacopeias, reishi is considered as an elixir for a wide variety of ailments (Sanodiya et al., 2009).

References to reishi as a superior herb that enhances human health can be found as early as 100 B.C. (Cao et al., 2012). Currently, members of the *G. lucidum* species complex continue to be prescribed in traditional medicine, for which fruiting bodies are typically grown, ground, and made into pills, tinctures, or teas (Stamets, 2000). In the *American Herbal Pharmacopeia, G. lucidum* sensu lato is mostly recommended for immune enhancing effects (Upton and Petrone, 2000; Jin et al., 2012). In addition, Eastern traditional medicine is becoming popular worldwide, and the reishi industry is quite profitable, with a world trade value of greater than $2.16 billion (Lai et al., 2004; Cao et al., 2012). The dietary supplement industry, which consists of vitamins, minerals, botanicals, etc., is a growing market with global sales at $109 billion, with an expectation of nearly doubling the sales by 2020 (Binns et al., 2017). Based on these figures, the reishi industry accounts for approximately 2% of the worldwide dietary supplement sales.

Recent research has reported that *G. lucidum* sensu lato contains approximately 400 bioactive compounds that are mostly polysaccharides and triterpenes (Sanodiya et al., 2009; Basnet et al., 2017). These compounds have anti-inflammatory, radical oxygen scavenging, anti-tumor, immune-enhancing, and antimicrobial activities (Paterson, 2006; Boh et al., 2007; Sanodiya et al., 2009; Jin et al., 2012). *G. lucidum* sensu lato produces the antifungal protein ganodermin, which has inhibitory effects against common fungi such as *Botrytis cinerea* and *Fusarium oxysporum* (Wang and Ng, 2006), and also produces other chemicals with antibacterial effects (Isaka et al., 2015; Basnet et al., 2017). Antiviral properties of triterpenes produced by another laccate species, *Ganoderma pfeifferi*, were shown to be active against influenza virus A (Mothana et al., 2003). Finally, nematacidal properties were observed when *Ganoderma* extracts were applied to *Heterodera glycines* (Zhao et al., 2009). In addition to these inhibitory effects against other microbes, reishi is mostly recommended for enhancing immunity (Upton and Petrone, 2000; Jin et al., 2012), with preventative qualities such as anti-inflammatory, anti-allergenic, radical oxygen scavenging, as well as inhibitory effects of tumor growths (Lin et al., 1991; Paterson, 2006; Powell, 2006; Boh et al., 2007; Joseph et al., 2009; Sanodiya et al., 2009; Jin et al., 2012). The chemical and biological properties of many fungi have sparked the interest of pharmaceutical researchers who are investigating secondary metabolites produced by fungi that may lead to the biomanufacturing of new drug formulations (Zhong and Xiao, 2009).

The taxonomy of the laccate *Ganoderma* species is quite convoluted, and the taxonomy and phylogenetic relationships among taxa are still being actively investigated (Moncalvo et al., 1995a; Hong and Jung, 2004; Cao et al., 2012; Wang et al., 2012; Zhou et al., 2015). For the past century, many studies of *Ganoderma* have used the name *G. lucidum* for any laccate *Ganoderma* species growing on hardwood trees (Pirone, 1957; Gilbertson and Ryvarden, 1986; Sinclair and Lyon, 2005; Zhou et al., 2015). Similarly, commercially available grow your own (GYO) reishi kits and vitamin supplements produced and marketed as traditional medicine are sold broadly as *G. lucidum.* Molecular studies have established that *G. lucidum* sensu stricto (Curtis) Karst has a native geographic distribution in Europe and some parts of China, while *Ganoderma lingzhi* Sheng H. Wu, Y. Cao, and Y.C. Dai is native from East Asia (Cao et al., 2012). Furthermore, as of this writing, *G. lucidum* sensu lato has been divided in to numerous distinct species (Welti and Courtecuisse, 2010; Cao et al., 2012; Wang et al., 2012; Zhou et al., 2015; Dai et al., 2017). The medicinal species most widely used is *G. lingzhi*, which has different morphological and genetic characteristics than *G. lucidum*.

Chemical constituents of mushrooms are generally expected to differ among species within a genus. For example, Kalogeropoulos et al. (2013) found significant differences among three species of *Lactarius* in production of sterols, phenolic acids, hydroxycinnamic acids, phenols, flavonoids, Stilbenes, and terpenic acids. In medicinal fungi, such as *Fomes fomentarius, Fomitopsis pinicola*, and *Piptoporus betulina*, individual isolates growing across different environments have also been shown to vary in the pharmacological constituents produced in fruiting bodies (Dresch et al., 2015). The chemical profiles of *G. lucidum* sensu stricto, a European species, and *G. lingzhi*, an Asian species, differ significantly in the amount of triterpenic acid produced in the basidiomata (Hennicke et al., 2016). Although a thorough analysis of chemical compositions has not yet been performed, it is probable that triterpenes and other bioactive chemical compositions vary across different phylogenetically supported *Ganoderma* taxa formerly included under the name *G. lucidum* (Hennicke et al., 2016).

In the United States, the Food and Drug Administration (FDA) does not regulate the marketing of fungal medicinal products. As with other herbal supplements, the lack of regulation can create a “buyer beware” market, because the integrity of the product lies with the manufacturer (Paterson, 2006; Raja et al., 2017). With the current rapidly changing understanding of *Ganoderma* characteristics among species, even well-intentioned manufacturers may have difficulty ensuring that their product contains the labeled species. Several Chinese species once called *G. lucidum* are now considered to belong to other species, including *Ganoderma flexipes, G. lingzhi, Ganoderma multipileum, Ganoderma sichuanense, G. sinense*, and *Ganoderma tropicum* (Wang et al., 2009, 2012; Cao et al., 2012; Hennicke et al., 2016; Raja et al., 2017). Surveys of consumer-relevant mushroom supplements have shed some light on the taxonomic identification of reishi; in a survey of multiple fungal supplement products, none of the six reishi products successfully tested with DNA barcoding methods contained *G. lucidum*, despite being labeled as “*G. lucidum*” (Raja et al., 2017). Furthermore, a study of chemical profiles identified by chromatography found that approximately 75% of reishi products sold in the United States are not consistent with the chemical profiles of *G. lucidum* sensu stricto (Wu et al., 2017). The goal of this research project was to survey what *Ganoderma* species were present in GYO kits and manufactured reishi products sold as dietary supplements, using traditional and next-generation DNA-based molecular techniques.

## Materials and Methods

### Sample Collection

Seventeen GYO kits that were marketed for medicinal use were purchased from mushroom cultivation companies in the United States. These kits were sold as colonized wooden dowels (*n* = 8), sawdust inoculum (*n* = 7), or mycelial suspensions in syringes (*n* = 2). All of the kits were labeled as containing *G. lucidum*, except one that was labeled as containing *Ganoderma curtisii.* However, one GYO kit was labeled with *G. lucidum* sensu lato, acknowledging the ambiguity of this species. Furthermore, two manufactured reishi products were labeled as containing a combination of fungal species: (1) *G. lucidum* and *Lentinula edodes*, or shiitake (product AL-R4) and (2) *Ganoderma tsugai* (presumably the improper spelling of *G. tsuage*), *G. lucidum*, and *G. applanatum* (product AL-R11). In addition, twenty commercially available, manufactured reishi products were purchased based on the following criteria: (i) readily available for purchase online, (ii) labeled as including *G. lucidum*, and (iii) marketed with medicinal benefits. Manufactured reishi products were sold as capsules (*n* = 13), powders (*n* = 3), tablets (*n* = 1), coffee (*n* = 1) or tea (*n* = 1). With the exception of two products labeled as *G. lucidum* sensu lato, acknowledging the ambiguity of the species *G. lucidum*, all products were labeled as “*G. lucidum*” with health-promoting marketing statements such as “supports longevity,” “supports immune system,” “detoxifier,” and “botanical immune support.” In addition, all manufactured products contained the following label: “*These statements have not been evaluated by the Food and Drug Administration. This product is not intended to diagnose, treat, cure or prevent any diseases.*”

*Culturing and vouchering GYO reishi kits*—Isolations from each GYO kit were made by excising small pieces (< 1 mm^3^) of spawn inoculum with a sterile scalpel and placing them onto 2% malt extract agar (MEA) (Difco Laboratories, Franklin Lakes, NJ, United States) prepared according to the manufacturer’s instructions with the addition of streptomycin (100 mg/l), 95% benomyl (4 mg/l), and 85% lactic acid (2 ml/l), which help to limit bacterial and the growth of fungi in the Ascomycota. Cultures were maintained on MEA slants as working stocks, and colonized agar disks were submerged in sterile water for long term storage (Marx and Daniel, 1976). Culture collections (ALM1-ALM17) were archived in the Center for Forest Mycology Research (CFMR) Culture Collection and Herbarium, USDA Forest Service, maintained by the Northern Research Station and housed in the Forest Products Laboratory, USDA-Forest Service in Madison, Wisconsin.

### Microscopic Analysis

The mycelium from the GYO kits was visualized in 5% KOH on glass slides using a Nikon Eclipse 55i light microscope (Melville, NY) to determine the presence/absence of chlamydospores, which are diagnostic features for some of the laccate *Ganoderma* species (Nobles, 1965; Hong and Jung, 2004). Two slide mounts were made for each GYO kit by taking mycelium from the center of the colony of a mature (eight-day-old) culture grown on MEA. These slides were visualized at 40× magnification, and scanned for the presence/absence of chlamydospores. Similarly, reishi supplement products were visualized on slide mounts with 5% KOH to look for any distinguishing microscopic features. Two slide mounts were made for each manufactured reishi products by crushing products (if necessary) and placing a small amount (< 1mg) of powder in a drop of KOH. Samples were then visualized at 40× by scanning each slide and thoroughly noting any features such as basidiospores, generative hyphae, skeletal hyphae, and chlamydospores. These features were used to deduce whether the products were made with immature basidiomata, mature basidiomata, vegetative cultures, or basidiospores.

### DNA Extraction, PCR, and Sanger Sequencing

DNA was extracted from each reishi supplement product and mycelium from GYO kit spawn with the Qiagen DNeasy Plant Mini Kit (Qiagen, Hilden, Germany) according to the manufacturer’s instructions. The internal transcribed spacer (ITS) region of the ribosomal DNA (rDNA) was amplified by PCR with primers ITS1F (5^′^ CTTGGTCATTTAGAGGAAGTAA) and ITS4b (5^′^ CAGGAGACTTGTACACGGTCCAG) (White et al., 1990; Gardes and Bruns, 1993) to identify *Ganoderma* species present in the sample. ITS is the universal fungal barcode for fungi that is commonly used for species delimitation (Schoch et al., 2012). In addition, the translation elongation factor 1-alpha (*tef1α*) was sequenced for all of the GYO kits using primers EF-Gano23F (5^′^ GGTGTCAGGCAGCTCATYGT) and EF-Gano887R (5^′^ CGAACTTGCARGCGATGTG), which were developed specifically for amplification of laccate *Ganoderma* species. For each PCR reaction, the following reagents were used: 12.5 μl of Immomix Red Master Mix (Bioline, London, United Kingdom), 8.5 μl of PCR-grade H_2_O, 1 μl BSA (20 mg/ml, Thermo Fisher Scientific, Waltham, MA, United States), 1 μl of each 10 mM primer, and 1 ng/μl of DNA template. Reactions were performed on a MJ Mini thermocycler (Bio-Rad, Hercules, CA, United States) with the following thermocycler conditions: cycle of 95°C for 10 min and followed by 35°cycles of 94°C for 30 s, variable annealing temperatures of 55°C (ITS) or 62°C (*tef1α*) for 30 s, and 72°C for 1 min, followed by a final extension step of 72°C for 5 min, and then 4°C. PCR products were visually assessed on a 1% agarose gel that was stained with Gel Red (Biotium, Fremont, CA, United States) to confirm successful amplification. Amplicons were purified with Exo-SAP-IT (Thermo Fisher Scientific, Waltham, MA, United States) according to the manufacturer’s recommendations. Sanger sequencing was performed using the same primers through the Genewiz^1^ sequencing lab. Forward and reverse sequences for each sample were aligned and visually edited using GENEIOUS 10 (Kearse et al., 2012). All Sanger sequences generated were deposited and accessioned in the GenBank sequence database (see below). ITS and *tef1α* sequences were queried against an in-house database of all known *Ganoderma* species (Zhou et al., 2015). Identifications were based on 99-100% homology with reliable reference sequences.

### Illumina Meta-Barcoding Sequencing

If multiple taxa were present in an individual sample, Sanger sequencing would either yield a clean DNA sequence only for the dominant taxon or would yield a mixed sequence that would not be readable due to multiple overlapping sequence peaks. Accordingly, we performed Illumina metabarcoding sequencing in addition to Sanger sequencing for all of the manufactured reishi products because these are the products that are most likely to contain multiple species based on the recommendation of Raja et al. (2017). DNA was extracted from the 20 manufactured reishi products as described previously, and all were subject to Illumina metabarcoding sequencing. Following extraction, DNA concentration was measured using a NanoDrop 2000 (Thermo Fisher Scientific, Waltham, MA, United States), and samples were equilibrated pre-PCR to 5 ng/μl. For each PCR reaction, the following reagents were used: 12.5 μl of Phusion High-Fidelity PCR Mix (New England Biolabs, Ipswich, MA, United States), 1.25 μl of each forward and reverse 5 μM primer, and 5–10 ng DNA. The ITS1 rDNA was amplified with fungal-specific primers ITS1f (Gardes and Bruns, 1993) and ITS2 (White et al., 1990) using eight i5 (forward) and three i7 (reverse) TruSeq barcoded adapters (Illumina, San Diego, CA, United States). PCR conditions were: denaturation at 94°C for 1 min followed by 30 cycles at 94°C for30 s, 52°C for 30 s, 68°C for 30 s and final extension at 68°C for 7 min, using 5–10 ng DNA. As a positive control, a six-species mock community was constructed using equimolar concentrations of DNA extracted from pure MEA cultures of GYO kits or wild collections. In addition, a negative PCR water control was used. Amplicons were verified on 1.5% agarose gels stained with SYBR Green (Invitrogen, Carlsbad, CA, United States) and normalized at equimolar concentration with the SequalPrep Normalization Plate Kit (Thermo Fisher Scientific, Waltham, MA, United States). If PCR bands were absent on the gel (*n* = 5, AL-R4, AL-R9, AL-R10, AL-R12, and AL-R19), samples were prepared as the other samples, except PCR products were not diluted. The library was purified with the Agencourt AMPure XP kit (Beckman Coulter, Brea, CA, United States) to remove primer dimers before sequencing with a MiSeq 300 bp paired-end protocol (Illumina, San Diego, CA, United States) at the Interdisciplinary Center for Biotechnology of University of Florida. Raw data are available at NCBI’s SRA BioProject Accession SRP149732.

### Illumina Meta-Barcoding Analysis

**FIGURE 1:**
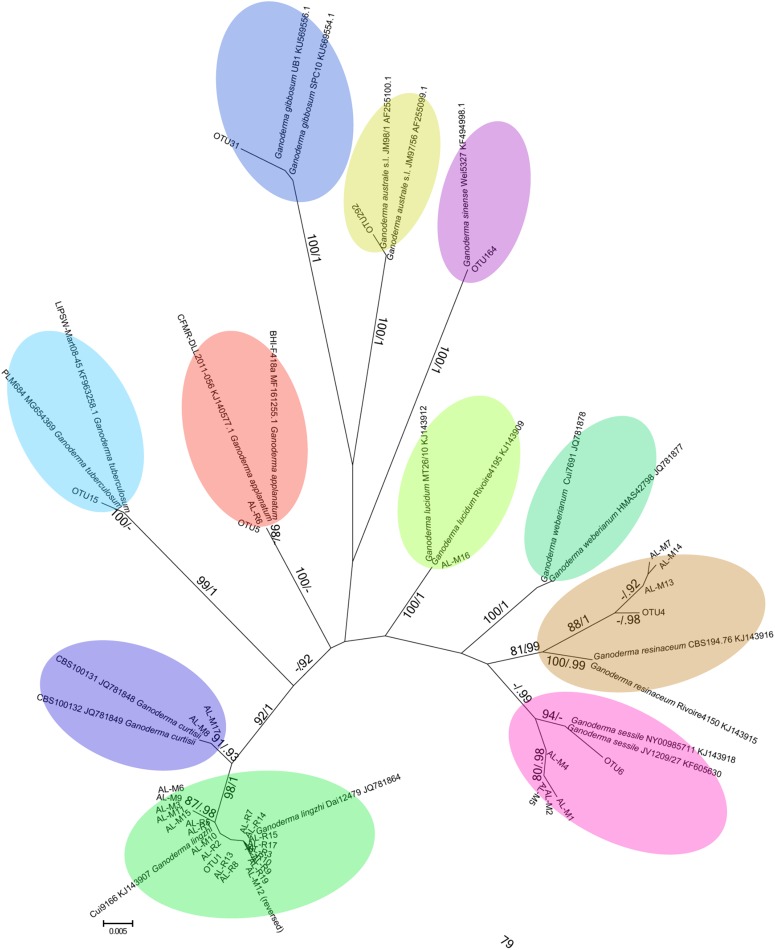
Unrooted maximum likelihood phylogenetic tree reconstructed using ITS sequences generated in this molecular survey together with reliable reference sequences for identification at the species level. Bootstrap values are indicated on branches, followed by the posterior probability of the Bayesian phylogeny that had identical topology. Reference sequences are annotated with species names and GenBank Accession numbers. Clusters of sequences shaded were found in this study, and sequence branches with the same color are the same species. Reference sequences for *G. weberianum* were included to show the relationship to the *G. resinaceum* sensu lato species identifications. Sequences generated with Sanger sequencing are labeled as AL-M# (GYO reishi kits) and AL-R# (manufactured products), and sequences generated with Illumina MiSeq are labeled as OTU# (manufactured products).

Illumina sequence data were processed using the AMPtk pipeline (version 1.1.0)^2^. Briefly, the overlapping 2 × 300 Illumina MiSeq reads were merged using USEARCH (version 9.2.64; Edgar and Flyvbjerg, 2015), all primers were removed from the merged reads. All reads that were less than 150 bp were removed. Reads that were less than 300 bp were padded with N’s, and those that were more than 300 bp were trimmed. This trimming/padding step improves clustering and other downstream steps (Palmer et al., 2018). Reads were then quality filtered with expected errors less than 1.0 (Edgar and Flyvbjerg, 2015), de-replicated, and clustered at 97% similarity, the broadly accepted cutoff to approximate species in fungi (Kõljalg et al., 2013), using UPARSE. All singleton OTUs were removed. The resulting OTU table was then filtered for index bleed at 0.5%, following Palmer et al. (2018). Taxonomy was assigned using the hybrid taxonomy approach in AMPtk. In order to verify the identities of the resultant OTUs, 60 ITS sequences that included reliable reference sequences as well as those generated from Sanger and Illumina sequences (**Table 1**) were aligned using the MAFFT (Katoh et al., 2002) plugin in GENEIOUS 10. The alignment was visually edited to remove any ambiguities and minimize differences that could have resulted from sequencing error. Visually edited alignments of each locus were used for independent phylogenetic analyses using maximum likelihood implemented in RAxML (Stamatakis, 2014) and a Bayesian inference using MrBayes (Ronquist et al., 2012) plugins in GENEIOUS 10. The RaxML analysis used a general time reversal (GTR) evolutionary model with rapid bootstrapping and 1000 bootstrap replications, and the Bayesian analysis used a GTR evolutionary model with a gamma rate variation using four gamma categories, for one million generations with 4 heated chains and a burn-in length of 100,000. These unrooted trees were produced to place ITS sequences and ITS1 OTUs generated from Sanger and Illumina sequencing into lineages identified based on reliable reference sequences (**Figure 1**). The alignments and trees have been deposited to Treebase under submission #22867.

**Table 1:** Sample labels, species, locations, product types and ITS GenBank Accession numbers for commercial reishi products and reference sequences used in the phylogenetic analysis.

Sample	Species^1^	GenBank ITS Accession^2^	Specimen Type or Location^3^	Authors^4^
AL-R6	*Ganoderma applanatum*	MH160077	Manufactured product	this study
BHI-F418a	*G. applanatum*	MF161255.1	MA, United States	Haelewaters et al., 2018
CFMR-DLL2011-056	*G. applanatum*	KJ140577.1	WI, United States	Brazee et al., 2014
OTU5	*G. applanatum*	NA	Manufactured product	this study
JM97/56	*Ganoderma australe* (species complex)	AF255099.1	NC, United States	Moncalvo and Buchanan, 2008
JM98/1	*G. australe* (species complex)	AF255100.1	NC, United States	Moncalvo and Buchanan, 2008
OTU292	*G. australe* (species complex)	NA	Manufactured product	this study
AL-M17	*Ganoderma curtisii*	MH160072	GYO Reishi Kit	this study
AL-M8	*G. curtisii*	MH160063	GYO Reishi Kit	this study
CBS100131	*G. curtisii*	JQ781848	NC, United States	Cao et al., 2012
CBS100132	*G. curtisii*	JQ781849	NC, United States	Cao et al., 2012
OTU31	*Ganoderma gibbosum*	NA	Manufactured product	this study
SPC10	*G. gibbosum*	KU569554.1	Brazil	Bolanos et al., 2016
UB1	*G. gibbosum*	KU569556.1	Brazil	Bolanos et al., 2016
AL-M10	*Ganoderma lingzhi*	MH160065	GYO Reishi Kit	this study
AL-M11	*G. lingzhi*	MH160066	GYO Reishi Kit	this study
AL-M12	*G. lingzhi*	MH160067	GYO Reishi Kit	this study
AL-M15	*G. lingzhi*	MH160070	GYO Reishi Kit	this study
AL-M3	*G. lingzhi*	MH160058	GYO Reishi Kit	this study
AL-M6	*G. lingzhi*	MH160061	GYO Reishi Kit	this study
AL-M9	*G. lingzhi*	MH160064	GYO Reishi Kit	this study
AL-R1	*G. lingzhi*	MH160073	Manufactured product	this study
AL-R10	*G. lingzhi*	MH160081	Manufactured product	this study
AL-R13	*G. lingzhi*	MH160082	Manufactured product	this study
AL-R14	*G. lingzhi*	MH160083	Manufactured product	this study
AL-R15	*G. lingzhi*	MH160084	Manufactured product	this study
AL-R17	*G. lingzhi*	MH160085	Manufactured product	this study
AL-R19	*G. lingzhi*	MH160086	Manufactured product	this study
AL-R2	*G. lingzhi*	MH160074	Manufactured product	this study
AL-R3	*G. lingzhi*	MH160075	Manufactured product	this study
AL-R5	*G. lingzhi*	MH160076	Manufactured product	this study
AL-R7	*G. lingzhi*	MH160078	Manufactured product	this study
AL-R8	*G. lingzhi*	MH160079	Manufactured product	this study
AL-R9	*G. lingzhi*	MH160080	Manufactured product	this study
Cui9166	*G. lingzhi*	KJ143907	China	Zhou et al., 2015
Dai12479	*G. lingzhi*	JQ781864	China	Cao et al., 2012
OTU1	*G. lingzhi*	NA	Manufactured product	this study
AL-M16	*Ganoderma lucidum*	MH160071	GYO Reishi Kit	this study
MT26/10	*G. lucidum*	KJ143912	Czech Republic	Zhou et al., 2015
Rivoire4195	*G. lucidum*	KJ143909	France	Zhou et al., 2015
CBS194.76	*Ganoderma resinaceum*	KJ143916	Netherlands	Zhou et al., 2015
Rivoire4150	*G. resinaceum*	KJ143915	France	Zhou et al., 2015
AL-M13	*G. resinaceum* s.l.	MH160068	GYO Reishi Kit	this study
AL-M14	*G. resinaceum* s.l.	MH160069	GYO Reishi Kit	this study
AL-M7	*G. resinaceum* s.l.	MH160062	GYO Reishi Kit	this study
OTU4	*G. resinaceum* s.l.	NA	Manufactured product	this study
AL-M1	*Ganoderma sessile*	MH160056	GYO Reishi Kit	this study
AL-M2	*G. sessile*	MH160057	GYO Reishi Kit	this study
AL-M4	*G. sessile*	MH160059	GYO Reishi Kit	this study
AL-M5	*G. sessile*	MH160060	GYO Reishi Kit	this study
JV1209/27	*G. sessile*	KF605630	AZ, United States	Zhou et al., 2015
NY00985711	*G. sessile*	KJ143918	NY, United States	Zhou et al., 2015
OTU6	*G. sessile*	NA	Manufactured product	this study
OTU164	*Ganoderma sinense*	NA	Manufactured product	this study
Wei5327	*G. sinense*	KF494998.1	China	Zhao and Cui, 2013
LIPSW-Mart08-45	*Ganoderma tuberculosum*	KF963258.1	French West Indies	Lesage-Meesen et al., 2014
OTU15	*G. tuberculosum*	NA	Manufactured product	this study
PLM684	*G. tuberculosum*	MG654369	FL, United States	Loyd, 2018
Cui7691	*Ganoderma weberianum*	JQ781878	China	Cao et al., 2012
HMAS42798	*Ganoderma weberianum*	JQ781877	China	Cao et al., 2012

*Authors of reference sequences are in the “authors” column. ^1^Species name based on reliable reference collection. ^2^ITS Accession numbers are given if produced with Sanger sequencing or from reference sequences. If sequence in tree was from metabarcoding the OTU data was deposited in the NCBI metabarcoding repository due to reads less than 300 bp, and the “NA” denotes “not applicable.” ^3^Sample location if it is a reference sequence, and sample type if it is one of the commercially available reishi products. ^4^Authors of the sequence from GenBank, unless published. If published, then the publication first author and year is cited.*

## Results

### Microscopic Analysis

Of the GYO kits, 100% of the pure cultures had generative hyphae with clamp connections and were consistent with *in vitro* colony morphology of *Ganoderma* (Nobles, 1965; Adaskaveg and Gilbertson, 1989). Forty-one percent (*n* = 7) of the seventeen GYO kits constitutively produced smooth, intercalary to terminal, double-walled, hyaline, ovate to obpyriform chlamydospores that are consistent with species in the *Ganoderma resinaceum* clade (Hong and Jung, 2004). No other diagnostic structures were observed in the vegetative mycelium of the other GYO kit products. Thirty-five percent (*n* = 7) of the manufactured reishi products were putatively made with mature fruiting bodies, based on the identification of generative and skeletal hyphae and basidiospores. Twenty-five percent (*n* = 5) were putatively made with immature fruiting bodies (generative and skeletal hyphae, no basidiospores), twenty percent (*n* = 4) were putatively made with cultures (generative hyphae only), and ten percent (*n* = 2) were putatively made with only basidiospores. Five percent (*n* = 1, AL-R15-coffee) of the manufactured products had no definitive *Ganoderma* tissues based on our slide mounts (**Table 2**).

**Table 2:** Summary of the Sanger sequencing results comparing the product (GYO reishi kits and manufactured reishi products) taxonomy labels to the DNA-based barcode identification.

Sample #^1^	Product^2^	Product Label^3^	Taxon Identified^4^	GB ITS/*tef1α* Accession
AL-M1_sd_	GYO reishi kit	*G. lucidum*	*G. sessile*_ch_	MH160056/MH168053
AL-M2_cd_	GYO reishi kit	*G. lucidum*	*G. sessile*_ch_	MH160057/MH168054
AL-M3_sy_	GYO reishi kit	*G. lucidum*	*G. lingzhi*	MH160058/MH168055
AL-M4_cd_	GYO reishi kit	*G. lucidum*	*G. sessile*_ch_	MH160059/MH168056
AL-M5_cd_	GYO reishi kit	*G. lucidum*	*G. sessile*_ch_	MH160060/MH168057
AL-M6_sd_	GYO reishi kit	*G. lucidum*	*G. lingzhi*	MH160061/MH168058
AL-M7_cd_	GYO reishi kit	*G. lucidum* s.l.	*G. resinaceum* s.l._ch_	MH160062/MH168059
AL-M8_cd_	GYO reishi kit	*G. lucidum*	*G. curtisii*	MH160063/MH168060
AL-M9_sd_	GYO reishi kit	*G. lucidum*	*G. lingzhi*	MH160064/MH168061
AL-M10_sd_	GYO reishi kit	*G. lucidum*	*G. lingzhi*	MH160065/MH168062
AL-M11_sy_	GYO reishi kit	*G. lucidum*	*G. lingzhi*	MH160066/MH168063
AL-M12_cd_	GYO reishi kit	*G. lucidum*	*G. lingzhi*	MH160067/MH168064
AL-M13_sd_	GYO reishi kit	*G. lucidum* s.l.	*G. resinaceum* s.l._ch_	MH160068/MH168065
AL-M14_sd_	GYO reishi kit	*G. lucidum* s.l.	*G. resinaceum* s.l._ch_	MH160069/MH168066
AL-M15_sd_	GYO reishi kit	*G. lucidum*	*G. lingzhi*	MH160070/MH168067
**AL-M16_cd_**	**GYO reishi kit**	***G. lucidum***	***G. lucidum***	MH160071/MH168068
**AL-M17_sd_**	**GYO reishi kit**	***G. curtisii***	***G. curtisii***	MH160072/MH168069
AL-R1_ca_	Manufactured product	*G. lucidum*	*G. lingzhi*_cu_	MH160073/-
AL-R2_ca_	Manufactured product	*G. lucidum*	*G. lingzhi*_mfb_	MH160074/-
AL-R3_ca_	Manufactured product	*G. lucidum*	*G. lingzhi*_cu_	MH160075/-
AL-R4_ca_	Manufactured product	*G. lucidum/Lentinula edodes*	FAILED_ifb_	-/-
AL-R5_ca_	Manufactured product	*G. lucidum*	*G. lingzhi*_mfb_	MH160076/-
AL-R6_ca_	Manufactured product	*G. lucidum*	*G. applanatum*_mfb_	MH160077/-
AL-R7_ca_	Manufactured product	*G. lucidum*	*G. lingzhi*_sp_	MH160078/-
AL-R8_ca_	Manufactured product	*G. lucidum*	*G. lingzhi*_ifb_	MH160079/-
AL-R9_po_	Manufactured product	*G. lucidum*	*G. lingzhi*_sp_	MH160080/-
AL-R10_po_	Manufactured product	*G. lucidum*	*G. lingzhi*_ifb_	MH160081/-
AL-R11_ca_	Manufactured product	*G. tsugai^5^, G. lucidum, G. applanatum*	FAILED_cu_	-/-
AL-R12_po_	Manufactured product	*G. lucidum*	FAILED_mfb_	-/-
AL-R13_ca_	Manufactured product	*G. lucidum* s.l.	*G. lingzhi*_cu_	MH160082/-
AL-R14_po_	Manufactured product	*G. lucidum*	*G. lingzhi*_sp_	MH160083/-
AL-R15_co_	Manufactured product	*Ganoderma sp.*	*G. lingzhi*_nd_	MH160084/-
AL-R16_ta_	Manufactured product	unknown	FAILED_mfb_	-/-
AL-R17_ca_	Manufactured product	*G. lucidum* s.l.	*G. lingzhi*_ifb_	MH160085/-
AL-R18_ca_	Manufactured product	*G. lucidum*	FAILED_ifb_	-/-
AL-R19_ca_	Manufactured product	*G. lucidum*	*G. lingzhi*_mfb_	MH160086/-
AL-R20_te_	Manufactured product	*G. lucidum*	FAILED_mfb_	-/-

*Products in bold were correctly labeled. ^1^Sample name with subscripts that code for type of product. For GYO kits (AL-M#), “cd” is for “colonized dowel,” “sd” is for “sawdust spawn,” and “sy” is for “syringe spawn.” For manufactured products (AL-R#), “ca” is for “capsule,” “po” is for “powder,” “ta” is for “tablet,” “co” is for “coffee,” and “te” is for “tea.” ^2^Products were either grow-your-own (GYO) reishi kits sold by mushroom cultivation companies in the United States or manufactured products, which were easily purchased online as capsules, powders, tablet, coffee, or tea. ^3^Product labels were the Ganoderma or relevant taxa that were written on the ingredients or nutritional label(s). If not present on label then product labels are coded as “unknown.” ^4^Taxon identified was based on Sanger sequencing with ITS and tefla for GYO reishi kits and ITS only for manufactured reishi products. If labeled with FAILED, then the reaction did not work. Subscripts indicate information about each product. For the GYO kits (AL-M#) “ch” is for “chlamydospores present.” For manufactured products “cu” is for “culture,” “ifb” is for “immature basidiomata,” “mfb” if for “mature basidiomata,” “sp” is for “only spores,” and “nd” is for “nothing detected.” ^5^Presumably, this product meant to have G. tsugae not G. tsugai.*

### Identification Based on Sanger Sequencing

ITS sequences were successfully amplified for 100% (*n* = 17) of the GYO kit samples and 70% (*n* = 14 of 20) of the manufactured reishi products. Sequences of *tef1α* were successfully generated for 100% (*n* = 17) of the GYO kit samples and amplification of the manufactured reishi products was not attempted. The 31 ITS sequences (MH160056-MH160086) and 17 *tef1α* sequences (MH168053-MH168069) were deposited in Genbank. Of the GYO kits, two products were correctly labeled as *G. curtisii* (AL-M17) and *G. lucidum* (AL-M16). The other 15 GYO kits were mislabeled as *G. lucidum*, and were actually identified as *G. lingzhi* (*n* = 7), *G. sessile* (*n* = 4), *G. resinaceum* sensu lato (*n* = 3), or *G. curtisii* (*n* = 1). All of the manufactured reishi products for which we generated sequences were mislabeled as *G. lucidum*, and subsequently identified with Sanger sequencing as *G. lingzhi*, except one sample (AL-R6) that was identified as *G. applanatum.* These results are presented in **Table 1**.

### Identification Based on Illumina Meta-Barcoding

**FIGURE 2:**
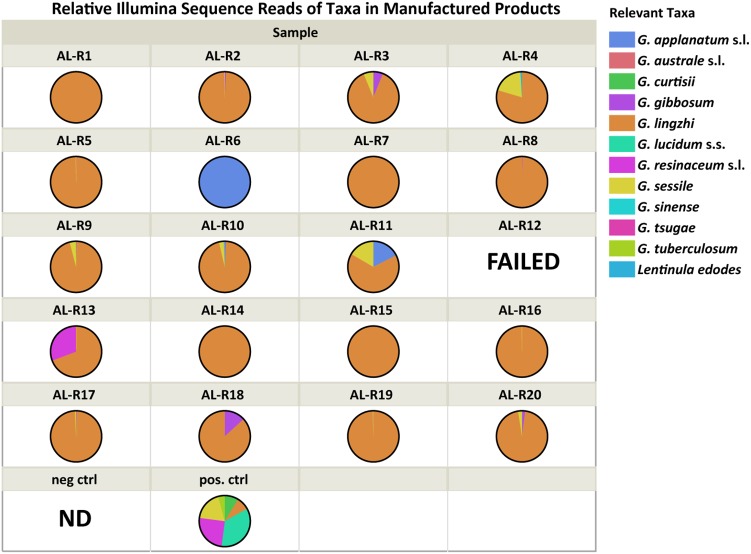
Relative Illumina sequence reads for each manufactured reishi product (i.e., dietary supplements). Each color represents a unique taxon that was either on the product label or a *Ganoderma* species detected with Next Generation Sequencing technology using the Illumina platform. “FAILED” means that the Illumina reaction did not yield good quality data. “ND” means nothing detected with the Illumina run/analysis.

Amplification and sequencing of the genomic library was successful for 19 of the 20 manufactured reishi products tested (AL-R12 failed). All successfully sequenced manufactured reishi products (*n* = 19) contained at least one *Ganoderma* species (**Table 3**). However, *G. lucidum* sensu stricto was not detected in any of the products. Three products each contained only a single *Ganoderma* species detected by Illumina meta-barcoding: *G. applanatum* in AL-R6 and *G. lingzhi* in AL-R7 and AL-R14 (**Figure 2**). We also detected multiple *Ganoderma* species in 16 of the 19 manufactured products. Because of PCR biases linked to next-generation sequencing methods, the quantification of species abundance in a community based on the abundance of sequences in a sample is not generally considered reliable (Palmer et al., 2018). However, we were able to detect the six species of our mock community in our positive control with relatively little bias (read numbers range from 6,015 to 51,020). In addition, our negative control yielded only one OTU that was not present in any of our samples, nor was a band observed of the PCR product with gel electrophoresis as described previously. We therefore used the relative abundance of sequences to quantify the presence of *Ganoderma* spp. in each sample (**Table 2**). The majority of products (74%, *n* = 14) contained a high abundance of *G. lingzhi* sequences, with only negligible numbers of sequences (< 5% of the reads) from other species of *Ganoderma* or other relevant taxa. Five out of the 19 successfully sequenced products had > 5% sequence abundance from other *Ganoderma* or relevant fungal species other than *G. lingzhi* (**Figure 2**).

**Table 3:** Illumina sequence reads for the manufactured products showing only the relevant taxa, which were either found on the label of the product or were *Ganoderma* species detected with the next generation sequencing technology using ITS1.

Sample #	Product Label^1^	Relevant Fungal Taxa Present Detected in Products^2^											
		*G. applanatum s.l.*	*G. australe*	*G. curtisii*	*G. lingzhi*	*G. lucidum s.s.*	*G. gibbosum*	*G. resinaceum s.l.*	*G. sessile*	*G. sinense*	*G. tuberculosum*	*Lentinula edodes*	*Total Reads^3^*
AL-R1	*G. lucidum*	0	0	0	27714	0	18	0	0	0	0	0	27732
AL-R2	*G. lucidum*	0	0	0	40336	0	356	0	0	0	0	0	40692
AL-R3	*G. lucidum*	0	0	0	2931	0	197	0	209	0	0	0	3337
AL-R4	*G. lucidum/L. edodes*	0	0	0	4290	0	0	0	1046	0	0	54	5390
AL-R5	*G. lucidum*	0	0	0	50463	0	161	0	0	0	0	0	50624
AL-R6	*G. lucidum*	21822	0	0	0	0	0	0	0	0	0	0	21822
AL-R7	*G. lucidum*	0	0	0	134057	0	0	0	0	0	0	0	134057
AL-R8	*G. lucidum*	0	0	0	4326	0	18	0	0	0	0	0	4344
AL-R9	*G. lucidum*	0	0	0	894	0	3	0	35	0	3	0	935
AL-R10	*G. lucidum*	0	0	0	11818	0	84	0	370	77	0	0	12349
AL-R11	*G. tsugai, G. lucidum, G. applanatum*	9243	0	0	34613	0	0	0	8729	0	0	0	52585
AL-R13	*G. lucidum s.l.*	0	0	0	32176	0	23	14193	46	0	0	0	46438
AL-R14	*G. lucidum*	0	0	0	85395	0	0	0	0	0	0	0	85395
AL-R15	*Ganoderma sp.*	0	0	0	40955	0	24	0	0	0	0	0	40979
AL-R16	*Ganoderma sp.*	0	0	0	67340	0	42	0	144	0	0	0	67526
AL-R17	*G. lucidum s.l.*	19	19	0	55720	0	239	0	353	0	76	0	56407
AL-R18	*G. lucidum*	0	0	0	1797	0	286	0	0	0	0	0	2083
AL-R19	*G. lucidum*	0	0	0	4407	0	8	0	17	0	0	0	4415
AL-R20	*G. lucidum*	0	0	0	27215	0	563	0	682	0	0	0	28460
Pos. Ctrl^4^	*G. curtisii, G. lingzhi, G. lucidum s.s., G. resinaceum s.l., G. sessile, G. tuberculosum*	0		12822	12099	51020	0	36548	27460	0	6015	0	145964
Total Reads^5^		31084		12822	638546	51020	2022	50741	39074	77	6094	54	831534
Total Reads in Product^6^		31084		0	626447	0	2022	14193	11614	77	79	54	685570

*^1^Product labels were the Ganoderma or relevant taxa that were written on the ingredients or nutritional label(s). ^2^Relevant taxa were those detected via Illumina sequencing method. ^3^Total Illumina sequence reads per the relevant taxa in each product. ^4^The positive control was a mock community spiked with equal volumes of each taxon at 5 ng/μl purified DNA. ^5^Total Illumina sequence reads for all products and positive control. ^6^Total Illumina sequence reads for all products minus the positive control.*

The maximum likelihood and Bayesian phylogenies constructed with sequences from Sanger and Illumina sequencing gave identical results, so only the unrooted RAxML phylogeny is presented (**Figure 1**). The ITS sequences generated in this study clustered with significant statistical support with the reference sequences.

## Discussion

The results of this study highlight the taxonomic problems surrounding the medicinal marketing of the laccate *Ganoderma* species that are used for cultivation spawn and/or sold as manufactured products (i.e., dietary supplements). The majority of the GYO kits and manufactured reishi products indicated that they were made with “*Ganoderma lucidum*.” However, no manufactured reishi products and only one GYO kit (AL-M16) actually contained *G. lucidum* sensu stricto. The incorrect identification of *Ganoderma* species in these products is presumed to be non-intentional and likely due to the complicated taxonomic problems within the laccate *Ganoderma* as well as the nomenclatural changes that have occurred in recent years (Cao et al., 2012; Wang et al., 2012; Zhou et al., 2015; Hennicke et al., 2016; Dai et al., 2017). In North America and around the world, many laccate *Ganoderma* species have been overlooked or treated as synonyms of *G. lucidum* for the past century. For example, the genome of *G. lucidum* that was sequenced as the model medicinal mushroom by Chen et al. (2012), is actually *G. lingzhi* based on the *rpb1* and *rpb2* DNA regions. Previous phylogenetic studies hypothesized that *Ganoderma* species have a vicariant pattern of evolution, wherein subclades typically contains sister taxa in North America and in Asia-Europe (e.g., *G. curtisii* and *G. lingzhi*) or geographically separated regions within North America (e.g., *Ganoderma tsugae* and *Ganoderma oregonense*) (Gilbertson and Ryvarden, 1986; Cao et al., 2012; Zhou et al., 2015; Hennicke et al., 2016). Based on these phylogenetic patterns, we expect that *G. lucidum* and other laccate *Ganoderma* species do not have a global distribution unless they have been introduced by humans. Yet the common practice for the past century has been to use the epithet “*G. lucidum*” for laccate *Ganoderma* species found on hardwoods across Asia, Europe, and North America. This cross-continental taxonomic lumping has made it impossible to draw clear inferences on the biology and chemistry of this culturally and ecologically important genus of wood decay fungi, as it is often unclear which species was under examination in any particular study. This taxonomic problem has been extended to the commercial medicinal fungi industry where products containing any laccate *Ganoderma* species are generally labeled as containing *G. lucidum* without additional information about the origin of the product or whether it was produced from multiple sources or species. The discovery of distinct species within the *G. lucidum* species complex has raised questions about inferences drawn in previous ecological studies of this genus (Loyd et al., 2018a,b). It also elucidates potential sources of variability in product chemistries and efficacies, and presents challenges for potential drug discovery efforts.

Of the 36 GYO kits and manufactured reishi products that were labeled as including *G. lucidum* in our survey, we found that 86% of them included a product substitution. Most of the time *G. lingzhi* was substituted for *G. lucidum.* As a medicinal product, this reishi “substitution” is probably more appropriate than the *G. lucidum* indicated on the label. *G. lingzhi*, which is native to East Asia, was recently circumscribed as one of the species previously mislabeled as *G. lucidum* sensu lato in Asia (Cao et al., 2012). *G. lingzhi* is likely the species that should properly be associated with the common names “reishi” and “lingzhi,” used in Chinese medicine (Dai et al., 2017). We also found evidence that other taxa were substituted for *G. lucidum*, including *G. applanatum* sensu lato, *G. australe* sensu lato, *G. curtisii, G. gibbosum, G. resinaceum* sensu lato, and *G. sessile.* These taxa were sold as GYO kits or produced > 5% of the Illumina sequence reads for an individual manufactured reishi product. All of these species are genetically distinct from *G. lucidum*, which is a species native to the temperate forests of Europe and some parts of China (Cao et al., 2012; Wang et al., 2012). These other species are also morphologically quite distinct from each other. In fact, *G. applanatum, G. australe*, and *G. gibbosum* have non-laccate (dull) pilei and belong to the subgenus *Elfvingia* (Moncalvo et al., 1995b). *G. curtisii* and *G. resinaceum* produce laccate (shiny) pilei as in *G. luccidum* but belong to two distinct subclades within the subgenus *Ganoderma* (Zhou et al., 2015). Based on the genetic diversity of the taxa sold as GYO kits and manufactured reishi products, it is likely that there are significant differences in the quality and quantity of medicinally relevant chemicals among the products being sold as “reishi,” and specified as “*G. lucidum*.”

Species substitutions in consumer products have been observed in other medicinal and culinary fungi as well. These include *Cordyceps, Boletus* and *Phellinus* species, among others (Dentinger and Suz, 2014; Raja et al., 2017). Raja et al. (2017) found that many of the products labeled with *Cordyceps sinensis*, a highly prized medicinal fungus, were identified based on DNA barcodes to *Tolypocladium inflatum* that belong to the same order of Hypocreales as *C. sinensis.* Similarly, Dentinger and Suz (2014) identified three novel species of *Boletus* in a single commercially sold packet of Chinese porcini mushrooms, assumed to be *Boletus edulis*, at a London supermarket. Furthermore, Newmaster et al. (2013) revealed that the herbal medicine industry (i.e., medicinal plant products) suffers from the same mislabeling problems. The authors showed that 68% of products tested (30 of 44) had species substitutions and about 33% of these products had fillers or contaminants that were not listed on the product label. Furthermore, some of the unlabeled contaminants/fillers were found to pose potential health risks to consumers (Newmaster et al., 2013). We detected a different species of *Ganoderma* than the one indicated on the product label in all but two of the products we tested. In a more limited sampling, Raja et al. (2017) also showed that reishi products labeled as *G. lucidum* were either *G. sichuanense* or *G. resinaceum. G. sichuanense* was circumscribed prior to *G. lingzhi*, but the holotype was not consistent with the original description of *G. sichuanense.* In fact, the holotype of *G. sichuanense* has morphology and ITS sequences that are more similar to *Ganoderma weberianum*. Furthermore, this confusion is exacerbated by the fact that the two names *G. lingzhi* and *G. sichuanense* continue to be used in the literature (Cao et al., 2012; Wang et al., 2012; Zhou et al., 2015; Hennicke et al., 2016; Dai et al., 2017; Raja et al., 2017) for the same species, despite the confusion arisen from the type specimens/sequences of *G. sichuanense.* As mentioned in previous studies (Cao et al., 2012; Zhou et al., 2015; Dai et al., 2017), we propose that the name *G. lingzhi* should be used until the taxonomy of *G. sichuanense* is resolved.

Wu et al. (2017) tested the quality of reishi supplement products by evaluating the polysaccharide and triterpene profiles using chromatography and saccharide mapping, and found that only 26.3% of the products tested were consistent with the triterpene and polysaccharide profile of an authenticated *G. lucidum* sensu stricto sample. Based on our results, most manufactured reishi products (i.e., dietary supplements), and nearly half of the products from the GYO kits sold in the United States contained the Asian species *G. lingzhi* despite being labeled as *G. lucidum.* Hennicke et al. (2016) showed that *G. lucidum* and *G. lingzhi* were not only genetically distinct based on the beta-tubulin gene, but were also chemically different. Extracts of *G. lingzhi* produced significantly more triterpenic acids than extracts made with *G. lucidum* sensu stricto (Hennicke et al., 2016). Although the products were mislabeled, current evidence suggests that *G. lingzhi* is the most widely used species in traditional Chinese medicine. More data are needed, however, to verify that other taxa were not used traditionally as well (Cao et al., 2012; Dai et al., 2017). In addition to *G. lingzhi*, the GYO kits were found to include *G. curtisii, G. lucidum, G. sessile*, and *G. resinaceum* sensu lato. Of these taxa, *G. curtisii* and *G. sessile* are native to the United States, whereas *G. resinaceum* and *G. lucidum* are native to Europe. Furthermore, *G. curtisii* is the North American sister taxon to the widely cultivated *G. lingzhi* (Zhou et al., 2015), and could potentially share similar ecological, biological and chemical properties. However, with the exception of *G. lucidum* sensu stricto, no study has evaluated the medicinal properties of these *Ganoderma* species, and how they relate to the widely cultivated *G. lingzhi* in Asia.

**FIGURE 3:**
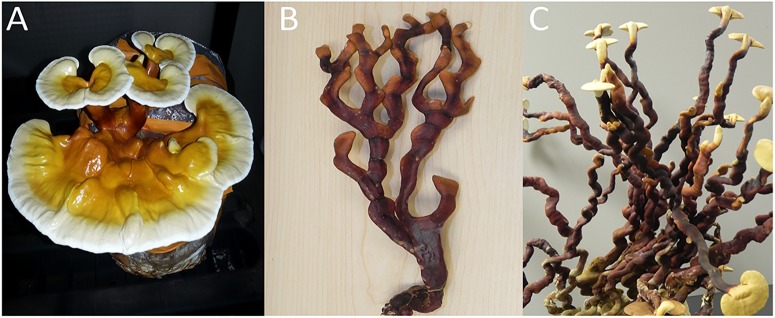
Fruiting bodies of *G. lingzhi* produced from GYO kits sold as *G. lucidum*. **(A)** “Kidney-shaped cap form” basidiomata, which is produced with good ventilation, **(B)** “antler form” basidiomata, which is produced under poor ventilation and high CO_2_, and **(C)** “anter form” basidiomata that transitioned into the “kidney-shaped cap form” after being exposed to better ventilation.

Our microscopic analyses indicated that in addition to species variability, companies are making manufactured reishi products out of various tissue types (e.g., basidiomata, mycelium, spores, etc.). Just as there is a dearth of research exploring biochemical differences among the newly elucidated *Ganoderma* taxa, few studies have investigated the differences in production of medicinally relevant chemicals produced within different tissue types. Heleno et al. (2012) investigated the differences in antioxidant potential of fruiting bodies, mycelium and spores of *G. lucidum* sensu stricto. The authors showed that phenolic compounds produced by *G. lucidum* had higher antioxidant potential than the polysaccharides (Heleno et al., 2012). Furthermore, extracts from fruiting bodies had the highest level of phenolics compared to the other tissue types (Heleno et al., 2012). Lastly, they found that mycelium had the lowest level of phenolics, but the highest level of polysaccharides (Heleno et al., 2012). Similarly, Sudheer et al. (2018) evaluated cultivation conditions (e.g., carbon dioxide concentration and light) that promote stipe elongation (“antler form”) vs. pileus formation (“kidney-shaped cap”) (**Figure 3**), and biochemical properties associated with each one of these unique isolate of *G. lucidum*. The “antler form” showed significantly more production of phenolics, flavonoids, polysaccharides and ganodermin, compared to the fruiting bodies that formed a true pileus (kidney-shaped cap form) (Sudheer et al., 2018). As pharmaceutical research continues to identify specific medicinally valuable compounds produced by *Ganoderma* species and characterize their biological activities, more research investigating the production of relevant compounds within each tissue type is warranted to improve product efficacy and consistency, and/or to lay the groundwork for the biomanufacturing of specific desirable compounds.

In addition to the medicinal relevance of the diverse *Ganoderma* species, there are many ecological implications in regard to cultivating non-native *Ganoderma* taxa outside of their native range. Of the 17 GYO kits purchased by companies in the United States, 11 purchased kits were of *Ganoderma* taxa that are not native to the United States. These taxa included *G. lingzhi* (native to Asia), *G. lucidum* (native to Europe and Asia), and *G. resinaceum* sensu lato (native to Europe). Previously, it was shown that monokaryotic isolates of the North American native *G. sessile* (previously considered in publications as *G. lucidum*) were compatible with monokaryotic European isolates of *G. resinaceum*, a species native to Europe (Adaskaveg and Gilbertson, 1986). Phylogenetic studies have shown that these species are sister taxa within the resinaceum subclade (Zhou et al., 2015). It is therefore possible that gene interchange could occur between related native and non-native *Ganoderma* taxa. When species are cultivated the potential for escape is heightened because large numbers of basidiomata are typically produced in a small area. The escape into the wild of billions of propagules (i.e., basidiospores) of a single genotype could create a genetic bottleneck in the wild populations. This would ultimately lead to a reduction of genetic diversity in wild populations. This phenomenon has been previously shown with the common button mushroom *Agaricus bisporus* in California, where cultivated genotypes have escaped cultivation and displaced native genotypes (Kerrigan and Ross, 1989; Kerrigan, 1995). A similar threat of dominance by a single cultivated genotype has been postulated for the shiitake industry in its native range in Asia (Hibbett and Donoghue, 1996). It is also possible that introduced species or hybrids between native and introduced taxa may be more aggressive wood decay fungi than the native species, which could have unintended consequences. For example, Coetzee et al. (2001) showed that a European genotype of *Armillaria mellea*, that was causing root rot on oaks and other woody shrubs, was introduced to South Africa over 300 years ago, presumably on nursery stock. More recently, the European species *Ganoderma adspersum* has been introduced to the San Joaquin Valley of California on almond root stocks, and is causing significant decay of the lower trunk/root flare resulting in tree failures due to windfall (Johnson, 2017). Moreover, in a recent survey of the laccate *Ganoderma* species in the United States, Loyd (2018) found two small geographically isolated populations of the European species *G. lucidum* sensu stricto that were presumed to be introduced through nursery stock or the medicinal fungus trade. Conservation studies focusing on the impacts of introduced decay fungi should be conducted to understand the ramifications of cultivating non-native *Ganoderma* and other commercially produced species in the United States.

Based on the Dietary Supplement Health and Education Act (DSHEA) of 1994, the FDA is not responsible for analyzing the contents of dietary supplements (Dodge et al., 2011). According to the DSHEA, the manufacturer is responsible for the safety and integrity of the product that it sells (Dodge et al., 2011). Given the difficulties in identifying and accurately naming specimens of laccate *Ganoderma*, blame cannot be placed on any one institution, whether it be the government, academia, growers, manufacturers, or distributors. However, in light of results from this study and others (Newmaster et al., 2013; Dentinger and Suz, 2014; Raja et al., 2017), the United States. Food and Drug Administration should reconsider the way it regulates the dietary supplement industry, especially medicinal herbs and fungi. In addition, there are few to no regulations on growing non-native *Ganoderma* species outside their native range. Until more research is conducted regarding the ecological ramifications of growing *Ganoderma* taxa outside their native range, *Ganoderma* cultivators should focus on growing regionally specific taxa to avoid any potential escapes with non-native taxa.

Our research shows that GYO kits and manufactured products (i.e., dietary supplements) marketed as *G. lucidum* contain multiple *Ganoderma* species. The fact that these products are inaccurately labeled and/or contain a mix of species, but are nonetheless sold for medicinal uses, raises questions regarding the authenticity of the fungal products used by this industry. Important questions are also raised such as: Do all *Ganoderma* species produce similar quality and quantities of medicinally relevant compounds? Can phylogenetic placement of a *Ganoderma* species predict the presence or effectiveness of medicinally relevant compounds? Are all *Ganoderma* preparations (e.g., basidiomata tissues, cultivated mycelium, spores) equally useful for medicinal purposes? Does growing non-native *Ganoderma* species present risks that they may displace native decay fungi or cause root rot or detrimental decay on native trees? These questions should be addressed in subsequent research focusing on the cultivation and manufacturing of reishi products.

## Author Contributions

The conception of the paper was a collaborative idea with AL, BR, MS, and JS. The work was funded by a grant received by AL, JS, and RB. The microscopy and molecular lab work was conducted as a collaborative effort by AL, CT, and MJ. The manuscript was written by AL. The manuscript was edited and reviewed by AL, BR, MJ, CT, MS, RB, and JS.

## Conflict of Interest Statement

The authors declare that the research was conducted in the absence of any commercial or financial relationships that could be construed as a potential conflict of interest.

## Footnotes

1 www.genewiz.com

2 http://amptk.readthedocs.io
